# Canadian perspectives in multiple myeloma on the use of steroids in clinical practice based on patient and healthcare provider interviews

**DOI:** 10.3389/fonc.2022.1061417

**Published:** 2022-12-08

**Authors:** Farah McKenzie, Gabriel Gazzé, Joanne Hewitt, Kari Kolm, Debra Pollock, Suzanne Rowland, Tina Crosbie

**Affiliations:** ^1^ BC Cancer – Prince George Centre, Prince George, BC, Canada; ^2^ Royal Victoria Hospital, McGill University Health Centre, Montreal, QC, Canada; ^3^ Hematology and Oncology Department, Cross Cancer Institute, Edmonton, AB, Canada; ^4^ Malignant Hematology Department, Hamilton Health Sciences, Hamilton, ON, Canada; ^5^ Pharmacy Department, Moncton Hospital, Moncton, NB, Canada; ^6^ Pharmacy Department, Princess Margaret Cancer Centre, University Health Network, Toronto, ON, Canada; ^7^ The Ottawa Hospital, Ottawa, ON, Canada

**Keywords:** multiple myeloma, adverse effects (AE), steroid management, corticosteroids, clinical practice, treatment recommendations, patient’s quality of life

## Abstract

Corticosteroid (steroid) medications are associated with challenging adverse effects that can negatively impact patient quality of life. However, owing to a long legacy of effective use in treatment protocols, they remain a cornerstone of multiple myeloma (MM) care. We conducted a roundtable with Canadian healthcare providers (HCPs) with diverse healthcare backgrounds and involvement in MM care as well as with patients with MM. Our goal was to develop clear guidance for steroid management aimed at improving patient quality of life, taking into account patient perspective and experiences with managing the disease. Our recommendations, which are based on the insights acquired from this discussion, can be categorized to the following areas: steroid prescribing, dosing, and modifications; managing adverse effects; and patient-HCP communication. These recommendations can be used by the entire multi-disciplinary hematology team to improve patient quality of life while being treated with steroid medication for multiple myeloma.

## Introduction

While steroid medications have been used in the treatment of multiple myeloma (MM) for over 50 years, for example the first use of dexamethasone took place in the late 1960s (1), their dosing has evolved over time, especially following the discovery of their impact on morbidity and mortality. Historically, MM treatment was limited to monotherapies, such as chemotherapy, autologous stem cell transplants, or pulses of high-dose steroids. Today, there are many more options available to treat MM, including agents such as immunomodulatory imide drugs (IMiDs), proteasome inhibitors (PIs), monoclonal antibodies (mAbs), antibody-drug conjugates, bispecific antibodies, chimeric antigen receptor T-cells (CAR-T) (2, 3). Typically, these medications are combined with steroids to improve their efficacy and promote synergistic effects. There are currently few steroid-free regimens (4), although more steroid-sparing approaches are being explored.

Steroid medications are effective anti-myeloma agents. Dexamethasone, for example, induces apoptosis in MM cells by mediating the activity of protein complexes such as NF-κB and mTOR, which regulate clinically significant pathways for aging, inflammation, and cancer (5). However, much remains to be understood about the intricate action of steroids in cells. Steroids are also associated with significant adverse events (**Table 1**) and an increased mortality that makes treatment adherence challenging for some. Common adverse events include, for example, hypertension, increased blood sugar levels, and insomnia (8), which adds further complexity in the management of the disease.

**Table 1:** Possible AEs associated with corticosteroid dexamethasone treatments.

Adverse effects (6, 7)	Examples	Frequency
Cardiovascular	Hypertension^a^	Common
	Swelling in the ankles and feet (fluid retention)	NA
Metabolic and endocrine	Weight gain^b^; increased blood sugar levels^b^, especially in people with diabetes; diabetes complications^c^	Common
Dermatology	Acne^a^	Common
	Bruising easily; impaired wound healing; increased hair growth	NA
Gastrointestinal	Gastritis^a^, hiccups^c^	Common
	Heartburn; increased appetite	NA
Musculoskeletal	Muscle weakness^b^	Uncommon
Neuropsychiatric	Mood swings^c^; irritability^c^	Common
	Difficulty sleeping (insomnia)^b^	Uncommon
	Difficulty maintaining focus; feelings of euphoria; increased energy	NA
Ophthalmologic	Cataracts^a^	Common
Infections and immune suppression	Pneumonia^b^	Common
Other	Dizziness^b^	Uncommon

Frequency of AEs were defined ascommon (1% ≤ frequency < 10%), uncommon (0.01% ≤ frequency < 1%), and NA (data not available or unknown) for lenalidomide treatment with low dose dexamethasone (a), lenalidomide with low dose following one unspecified prior therapy (b) and dexamethasone alone (c) (8).

Patients taking steroid medications can benefit from the expertise and care provided by members of a multidisciplinary haematology team because effective treatment of steroid-associated adverse events can improve steroid adherence and treatment outcomes. Prompt management of patient concerns can contribute to successful treatment of MM (6).

This article aims to address the lack of clear direction on best practices with steroid management in MM, with the goal of improving the patient’s quality of life. This article aligns with Myeloma Canada’s 2021 top ten priorities (9) that include topics such as personalized medicine; safe reduction, cycling, and cessation of anti-myeloma medications in order to minimize side effects; and reduction and management of short and long-term adverse effects (AEs) of myeloma treatment.

We will present insights on steroid use in the management of MM emerging from in-depth consultations with two patients with MM as well as their participation in a roundtable discussion with diverse healthcare providers (HCPs). The latter included nurse practitioners, registered nurses, and pharmacists, with each member providing a different perspective on the issue. Several main themes emerged throughout the discussion. Based on the insight from the HCP discussion, we have created a set of recommendations designed to improve patient experience and reduce the impact of the AEs of steroids. We will then discuss implications of this initiative.

## Use of steroids in multiple myeloma management: Prescribing, dosing and modifications

Dexamethasone is the most commonly used steroid in the treatment of MM. Historically, dexamethasone was used as a standalone treatment, but is now often an adjunct to novel treatment regimens.

A variety of factors are used to establish the starting dose of steroids. Patient factors such as age, medical comorbidities, relative fitness, and goals of care influence steroid dosing. In patients who struggle with steroid-related AEs, the dose can be modified using a variety of strategies. Strategies may include reducing the total weekly dose, splitting the dose, or substituting for a different type of steroid medication altogether.

For example, in an elderly patient with multiple medical comorbidities, rather than starting at the standard dexamethasone 40 mg weekly, the provider may recommend a more conservative starting dose of less than 20 mg. In another example, a patient who struggles with agitation or irritability, with higher pulse doses, may better tolerate weekly dexamethasone dosage to be split into 2-3 days. In some instances, patients are unable to tolerate dexamethasone at all, in particular elderly patients (10), who may then be trialled with a different steroid such as prednisone (e.g., 2 mg/kg daily for 4 days every 4 weeks).

When prescribing steroids for MM, HCPs typically aim for the lowest effective dose for the minimum required time. Modifications may include dosage reduction or splitting of the dosage over two days instead of one in order to minimize AEs. HCPs agreed it was important to regularly re-evaluate and adjust dosage as required to minimize toxicities. They expressed the importance of caution in certain populations, such as when treating patients with a history of psychosis or dealing with elderly patients.

Tapering of the steroid dose was not identified as common practice among HCPs due to the once-weekly or pulse dosing still used in some scenarios or in pre-autologous stem cell transplant. However, doses can be tailored down to address tolerance issues. Steroid withdrawal may be of concern but tapering, if necessary, can be individually tailored.

Overall, HCPs agreed that their guiding principle was to try to maximize the positive, disease reducing effects of the steroid medication while minimizing the negative or AEs of this class of medication and trying to improve the patient experience.

While HCPs also acknowledged the efficacy and proven track record of steroid medications, they expressed interest in steroid-sparing or steroid-limited treatment approaches. A recent trial, for example, supported the feasibility of steroid-sparing by showing that lenalidomide (R) monotherapy was comparable to lenalidomide and dexamethasone (Rd) in terms of event-free survival in elderly patients (11). In this trial, patients receiving lenalidomide monotherapy also reported less AEs. Another clinical trial is currently underway at the University of Rochester to further investigate the efficacy of lenalidomide monotherapy (12). Similarly, reduction in dose intensity of the RVD regimen (lenalidomide, bortezomib, and dexamethasone), called RVD lite, in older transplant-ineligible newly-diagnosed MM patients (median age of 73 years) showed comparable efficacy and improved tolerability (13). However, more trial data is required to support lower doses, shorter duration of steroid use, and treatment combinations that do not include steroids in practice.

Recommendations for steroid dosing:

- Assess the patient’s baseline comorbidities, treatment preferences, and goals to adjust dose titration.
- If AEs are significant, then consider:
  o splitting or reducing the dose,
  o reducing the dose frequency,
  o substituting with another steroid.
- Discuss with the HCP and healthcare team to decide how to manage AEs (e.g., safety of modifying, reducing, or omitting a dose).
- Consider steroid-sparing based on results from ongoing research (12).

## Managing adverse effects: Screening and clinical best practices

The patients included in this roundtable expressed both psychosocial and physical health concerns with steroid treatment. Mental health concerns included emotional “highs and lows”, insomnia, aggression, difficulty functioning on dose day (with patients experiencing anxiety or excessively high energy) and the following day (with patients expressing they experienced an emotional crash). Concerns related to patients’ physical health included gastrointestinal troubles, such as heartburn, and ophthalmological concerns, such as cataracts. These concerns were specific to the two patients interviewed, but they help illustrate the kinds of issues patients who are treated with steroids may have.

HCPs emphasized the importance of knowing which AEs to monitor and agreed on several screening tools and other best practices to reduce AEs (**Table 2**). Patients shared their experience with respect to the most concerning or problematic steroid related AEs they encountered. HCPs agreed that they should be aware of their patients’ perspectives on their AEs resulting from steroid treatment. The goal should be to improve quality of life, and this may involve steroid dose changes, side-effect management, screening, and counselling. Improving quality of life is an ongoing conversation between patient and HCP.

**Table 2:** Recommendations to support AE management with steroid use.

Recommendations	Note
1. Partner with primary health care provider to manage the steroid effects on chronic diseases.	Chronic diseases include diabetes, osteoporosis, depression, anxiety, or substance use disorder. This is particularly important if considering prescribing drugs with misuse potential.
2. Manage the risk of gastrointestinal effects (e.g., heartburn, gastritis, and cramps).	Encourage patient to take steroid with food and avoid non-steroidal anti-inflammatory drugs (NSAIDs). If patient still experiences stomach upset, providers should then consider prescribing a proton pump inhibitor (PPI) or a H2-receptor antagonist (H2RA), which may only be required on steroid dose day.
3. Monitor blood glucose and bone density.	To monitor changes, HCPs recommend establishing baseline levels of HbA1c and baseline scores for fracture risk assessment (FRAX), bone mineral density (BMD) and Canadian Association of Radiologists and Osteoporosis Canada (CAROC) before starting treatment.
4. Consider non-pharmacotherapies to help with insomnia.	Non-pharmacotherapies include referral to counseling, sleep hygiene, and timing of steroid dose. Only then, should pharmacotherapies be considered. There are several options for dose timing, and different patients prefer different approaches. o Due to the insomnia it can cause, most patients take dexamethasone early in the day (e.g., with their breakfast) to allow the energy burst to wear off by bedtime. o Some patients choose to take steroids at bedtime with a snack as they find they can sleep for a few hours before the jitteriness and increased energy kicks in.
5. Apprise patients on the lows they may experience with steroids.	Strategies should be discussed in advance on how to recognize decrease in mood due to steroid use, have a prepared action plan prior to the reduction in mood, and to refrain from making important life decisions during those times.
6. Prevent infections.	Ensure up-to-date immunizations. In patients with long-term continuous dosing, antibiotic prophylaxis may be considered to prevent infection.
7. Ensure regular ophthalmologic or optometric exams.	These appointments are important to monitor for potential cataracts and other vision changes.
8. Consider consulting with specialists as needed.	For example, endocrinologist, ophthalmologist, or psychiatrist.

## Patient-HCP communication: A multidisciplinary effort and ongoing conversation

Patients and HCPs alike stressed the importance of ongoing and open communication surrounding steroid use between the patient and members of the care team. HCPs expressed that each member of the multidisciplinary team plays a role in ongoing assessment, counselling, education, and addressing patients concerns with steroids. Participants agreed that communication was best framed as an ongoing conversation, rather than something that can be accomplished in one appointment. Both patients stressed the importance of including their family and caregivers in conversations about steroid side-effects, especially regarding the psychological ones. Both patients and HCPs suggested speaking with spouses, partners or support people individually as they may express their observations and concerns more freely than when the patient is present.

Patients agreed that the volume of information about potential side-effects and their management for a myeloma regimen was a lot to take in at one time. They expressed a desire for clear, easy-to-understand educational material. Proposed ideas included a one-page printout or short video specific to steroid use. Patients also highlighted the importance of educating and including family and caregivers about steroid side-effects too. Patients agreed on the importance of self-advocacy and the benefit of support groups.

HCPs also shared talking points and tips to communicate with patients on steroid use in the management of MM.

Recommendations for communicating with patients on steroids:

- Organizations should focus on developing educational resources about steroid management such as one-page printouts and short videos.
- Continuously reinforce information about steroid use and treatment expectations:
  o Ensure ongoing conversation over the course of many appointments,
  o Consider follow-ups by phone and offer to answer questions,
  o Provide refresher, especially upon starting a new regimen.
- Have a patient-centered approach that highlights their preferences, lifestyle and goals of care.
- It is extremely important to include a patient’s family, especially their spouse, partner, or their caregiver in discussions – to ensure a complete understanding.
  o Providers might like to speak to a patient’s spouse one-on-one where possible to encourage a safer space to confide about the effects of the medication.
  o Family counselling can be helpful for some patients and loved ones.
- Patients should be encouraged to seek the help of a support group and should be referred to nationally and provincially recognized support groups, such as Myeloma Canada (14), whenever possible. This can include virtual or in-person support groups.

## Discussion

Based on a roundtable discussion with patients with MM and HCPs of diverse backgrounds, and an interview with two patients with MM treated with dexamethasone on a long-term basis, we have presented a set of recommendations for the use of steroids in the treatment of MM. The themes included patient-specific dosing modification, communication between provider and patient, and AE screening and management. The aim was to address a lack of clear guidance on steroid management in MM by producing actionable recommendations based on insights generated from productive conversation.

Patients expressed significant concerns and a reduced quality of life on steroids. Maintaining quality of life is an important goal for future treatment protocols. Our recommendations have been designed to help provider-patient partnerships maximize quality of life. While treatment protocols in the future may rely less heavily on steroid medications, they remain a cornerstone of MM treatment, and steroid management remains clinically relevant in the treatment of MM. It is our hope that these recommendations will provide much-needed clarity to diverse healthcare providers about best practices for steroid management in multiple myeloma.

## Data availability statement

The original contributions presented in the study are included in the article. Further inquiries can be directed to the corresponding author.

## Author contributions

All authors contributed equally to the conception, writing, and reviewing of this manuscript.

## References

[1] Alexanian R Haut A Khan AU Lane M McKelvey EM Migliore PJ . Treatment for multiple myeloma. combination chemotherapy with different melphalan dose regimens. Jama (1969) 208(9):1680–5. doi: 10.1001/jama.208.9.1680 5818682

[2] Ghosh N Ye X Ferguson A Huff CA Borrello I . Bortezomib and thalidomide, a steroid free regimen in newly diagnosed patients with multiple myeloma. Br J Haematol (2011) 152(5):593–9. doi: 10.1111/j.1365-2141.2010.08534.x PMC3412295 21241279

[3] Shah N Aiello J Avigan DE Berdeja JG Borrello IM Chari A . The society for immunotherapy of cancer consensus statement on immunotherapy for the treatment of multiple myeloma. J Immunother Cancer (2020) 8(2):1-28. doi: 10.1136/jitc-2020-000734 PMC7359060 32661116

[4] Burwick N Sharma S . Glucocorticoids in multiple myeloma: Past, present, and future. Ann Hematol (2019) 98(1):19–28. doi: 10.1007/s00277-018-3465-8 30073393

[5] Rosenberg AS . From mechanism to resistance - changes in the use of dexamethasone in the treatment of multiple myeloma. Leuk Lymphoma (2022) 28:1–9. doi: 10.1080/10428194.2022.2136950 36308022

[6] Faiman B Bilotti E Mangan PA Rogers K Board IMFNL . Steroid-associated side effects in patients with multiple myeloma: Consensus statement of the imf nurse leadership board. Clin J Oncol Nurs (2008) 12(3 Suppl):53–63. doi: 10.1188/08.CJON.S1.53-62 18490257

[7] Yasir M Goyal A Sonthalia S . Corticosteroid adverse effects. statpearls. Treasure Island (FL: StatPearls Publishing LLC (2022). 30285357

[8] Product monograph: “PrAPO- lenalidomide”. (Health Canada: Apotex Inc) (2021).

[9] Fowler S McLaughlin L Bridges S Robichaud M Ridgway B Reece D . The future of myeloma research in Canada and beyond: Results of a James Lind alliance priority setting partnership. Br J Haematol (2022) 196(5):e52–e4. doi: 10.1111/bjh.17946 PMC9135117 34741302

[10] Ludwig H Hajek R Tothova E Drach J Adam Z Labar B . Thalidomide-dexamethasone compared with melphalan-prednisolone in elderly patients with multiple myeloma. Blood (2009) 113(15):3435–42. doi: 10.1182/blood-2008-07-169565 18955563

[11] Larocca A Bonello F Gaidano G D’Agostino M Offidani M Cascavilla N . Dose/Schedule-adjusted Rd-r vs continuous Rd for elderly, intermediate-fit patients with newly diagnosed multiple myeloma. Blood (2021) 137(22):3027–36. doi: 10.1182/blood.2020009507 33739404

[12] Steroid sparing treatment with in newly diagnosed transplant ineligible patients with multiple myeloma. Available at: https://ClinicalTrials.gov/show/NCT04635189.

[13] O’Donnell EK Laubach JP Yee AJ Chen T Huff CA Basile FG . A phase 2 study of modified lenalidomide, bortezomib and dexamethasone in transplant-ineligible multiple myeloma. Br J Haematol (2018) 182(2):222–30. doi: 10.1111/bjh.15261 PMC6074026 29740809

[14] Myeloma Canada: National charitable organization (2022). Available at: www.myelomacanada.ca.

